# From Phytochemicals to Physiology: The Metabolic and Redox Effects of Botanical Extracts on Crops

**DOI:** 10.3390/plants15081237

**Published:** 2026-04-17

**Authors:** Fabián Pérez-Labrada, Antonio Juárez-Maldonado, Paola Fincheira, Froylán Rincón-Sánchez, Gonzalo Tortella, Susana González-Morales, Adalberto Benavides-Mendoza

**Affiliations:** 1 Departamento de Botánica, Universidad Autónoma Agraria Antonio Narro, Saltillo 25315, Mexico; fabperlab@outlook.com (F.P.-L.); juma841025@gmail.com (A.J.-M.); 2 Laboratorio Nacional CONAHCYT de Ecofisiología Vegetal y Seguridad Alimentaria (LANCEVSA), Universidad Autónoma Agraria Antonio Narro, Saltillo 25315, Mexico; 3 Centro de Excelencia en Investigación Biotecnológica Aplicada al Medio Ambiente (CIBAMA), Universidad de La Frontera, Temuco 4811230, Chile; paola.fincheira@ufrontera.cl; 4 Departamento de Fitomejoramiento, Universidad Autónoma Agraria Antonio Narro, Saltillo 25315, Mexico; frincon@uaaan.edu.mx; 5 SECIHTI-UAAAN, Saltillo 25315, Mexico; qfb_sgm@hotmail.com; 6 Departamento de Horticultura, Universidad Autónoma Agraria Antonio Narro, Saltillo 25315, Mexico

**Keywords:** biostimulants, plant extracts, medicinal plants, plant stress, oxidation-reduction metabolism

## Abstract

Botanical extracts have emerged as promising biostimulants in agricultural systems because of their ability to modulate key metabolic and redox processes in crops, thereby increasing stress tolerance and productivity. This review synthesizes current knowledge on how botanical extracts influence plant metabolism and redox homeostasis, with a particular emphasis on their role in adaptive cellular responses. Evidence indicates that these extracts can increase antioxidant enzyme activity, regulate reactive oxygen species (ROS) signaling, and promote the accumulation of bioactive metabolites associated with improved stress tolerance and enhanced growth. This review also examines how agronomic practices, including nutritional management, water availability, light regimes, and preharvest biostimulant applications, together with emerging biotechnological approaches, can be strategically employed to optimize the bioactive composition and efficacy of botanical extracts. By integrating recent advances in metabolomics and transcriptomics, the manuscript highlights the biochemical and molecular reprogramming triggered by botanical extracts. It identifies key challenges, including variability in extract composition, lack of standardization, and context-dependent responses. Finally, future research directions are outlined, emphasizing the need for mechanistic understanding, quantitative evaluation of plant responses, and the development of standardized frameworks to support the sustainable application of botanical extracts in agriculture.

## 1. Introduction

Life is fundamentally governed by redox reactions, which sustain metabolic processes and enable organisms to respond to environmental fluctuations. In plants, the redox potential gradient driving metabolism is primarily generated by photosynthesis, in which reducing equivalents derived from water establish a thermodynamic imbalance relative to oxygen [1,2]. Redox homeostasis, also referred to as redoxtasis [3], encompasses the coordinated regulation of reactive species and antioxidant systems, as well as the flow of reducing equivalents toward oxidation sinks [4]. Reactive species arise from electron-transfer reactions associated with photosynthetic and respiratory electron transport, as well as metabolic pathways involving glutathione, ascorbate, and other redox-active metabolites [5]. In plant cells, these species act not only as metabolic byproducts but also as key signaling molecules that regulate development and stress responses, with their levels tightly controlled by enzymatic and nonenzymatic antioxidant systems [6].

Failure to maintain redox homeostasis causes oxidative stress [7], which is defined as “an imbalance between oxidants and antioxidants in favor of the oxidants, potentially leading to damage” [8].

**Figure 1:**
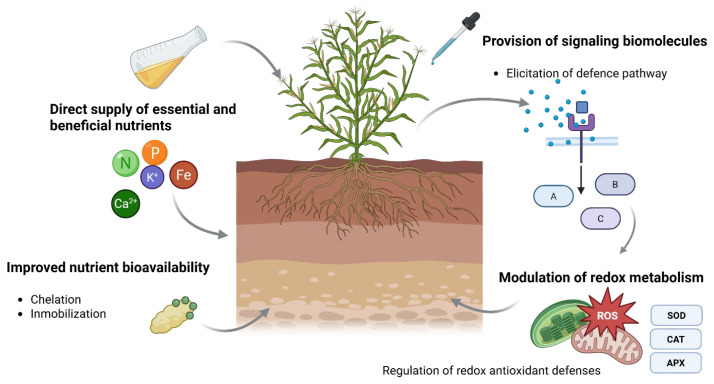
Mechanisms through which biostimulants enhance plant performance. Biostimulants exert their effects through (i) the direct supply of essential and beneficial nutrients (e.g., N, P, K, Mg, and Fe); (ii) improved nutrient bioavailability via processes such as chelation and mobilization; (iii) the provision of signaling biomolecules that activate defense pathways; and (iv) the modulation of redox metabolism through the regulation of antioxidant systems in key organelles such as chloroplasts and mitochondria (e.g., SOD, CAT, and APX), contributing to cellular homeostasis and stress tolerance.

Oxidative stress is associated with environmental stress, and in recent decades, biostimulants have emerged as valuable alternatives for mitigating stress in crops [9]. Biostimulants are defined as “a formulated product of biological origin that improves plant productivity as a consequence of the novel, or emergent properties of the complex of constituents, and not as a sole consequence of the presence of known essential plant nutrients, plant growth regulators, or plant protective compounds” [10]. Biostimulants can be classified into physical, chemical, and biological types [11]. As shown in Figure 1, biostimulants have beneficial effects on plants through various mechanisms, including direct supply of essential nutrients, improved nutrient bioavailability, the provision of signaling biomolecules that trigger defense responses, and modulation of redox metabolism [6,9].

Among biostimulants, botanical extracts, which are manufactured from the biomass of terrestrial plant species, stand out for their complex composition, including secondary metabolites, polysaccharide oligomers, peptides, amino acids, osmolytes, and various minerals [12,13]. Given the great diversity of constituents in a botanical extract, exploring how this compositional complexity influences plant redox modulation and how it can be strategically manipulated is relevant. Recent studies have emphasized that the biological activity of botanical extracts is strongly dependent on their chemical composition and the interactions among their constituents [13]. In this context, extraction conditions (e.g., solvent polarity and temperature), fractionation processes, and biotechnological approaches can selectively enrich specific bioactive compounds, including phenolics and bioactive peptides [14]. For instance, extracts enriched in phenolic compounds or essential oils have been associated with increased antioxidant capacity and the modulation of reactive oxygen species (ROS)-related metabolism in plants [15].

Several reviews on the agricultural use of botanical extracts have been published in recent years [16,17,18,19,20,21,22]. However, these studies generally address plant responses in broad terms, with limited emphasis on the underlying metabolic and redox mechanisms. In contrast, this review focuses specifically on the role of botanical extracts in modulating cellular redox homeostasis and associated metabolic pathways. Additionally, it highlights how agronomic practices, such as nutrient management, water availability, and light conditions, can influence the composition and efficacy of botanical extracts, providing a more applied and integrative perspective.

The objective of this review is to examine the impact of botanical extracts on adaptive cellular processes, with a particular focus on metabolic and redox mechanisms. It is intended as a resource for researchers exploring the underlying mechanisms of action of biostimulants. To ensure a comprehensive and balanced synthesis, the literature included in this review was selected through searches in major scientific databases (e.g., Web of Science, Scopus, and Google Scholar) using combinations of keywords such as botanical extracts, biostimulants, plant stress, redox metabolism, and antioxidant responses. Priority was given to peer-reviewed articles published in the last 10–15 years, although earlier seminal studies were included when relevant to conceptual development. Studies were selected on the basis of their direct relevance to plant physiological, metabolic, and molecular responses to botanical extracts. Reports focused exclusively on medical or nonplant systems were considered only when they provided mechanistic insights applicable to plant biology. This selection aimed to capture both consistent patterns and context-dependent responses across different crop systems and experimental conditions.

## 2. Botanical Extracts and Bioactive Constituents Relevant to Redox Responses

Owing to their origin, botanical extracts are complex biostimulants. It is challenging to determine the quantities of different components in a botanical extract, as their compositions depend on the measurements made by the study authors. The inherent complexity can also be increased by the interactions of the plants that produce the biomass from which the botanical extract is obtained with their environment, making it difficult to obtain a botanical extract of uniform composition [23]. This lack of compositional uniformity represents a major limitation for reproducibility and hampers the establishment of clear cause-and-effect relationships between extract composition and plant responses. Therefore, a practical approach is to standardize botanical extracts by selecting a representative compound or group of compounds as a reference marker. In this context, phenolic content is commonly used because of its strong association with antioxidant capacity and redox-related biological activity [24]. Indeed, several studies have shown that standardization based on total phenolic content improves the consistency of biological responses, particularly antioxidant activity and the modulation of reactive oxygen species (ROS) metabolism, thereby facilitating reproducibility and comparisons across different plant-derived formulations [14,24].

The first determinant of the composition of the botanical extract is taxonomy. Even closely related varieties within the same species can exhibit significant differences. For example, the aromatic herbs of the *Ocimum basilicum* species present distinct chemotypes. The variety Sweet Dani accumulates citral (neral and geranial), whereas the variety Cinnamon accumulates linalool and (E)-methyl cinnamate-rich profiles [25]. Interestingly, adequate fertilization and growth under protected conditions increased biomass productivity without affecting essential oil content; similarly, *Mentha spicata* and *M. longifolia* ssp. Compared with pulegone and 1,8-cineole, which are dominant in other species, Cyprica differs markedly in its carvone and limonene contents [26]. These examples highlight that even subtle genetic or agronomic differences can lead to substantial variation in phytochemical profiles, which in turn may result in divergent biostimulant effects across studies.

The second determinant of the composition of botanical extracts is the impact of environmental factors on gene expression and the modulation of metabolic pathways. Intrinsic species differences define the profile and the maximum attainable pool of secondary metabolites. Nevertheless, the realized composition is highly plastic and is modulated by environmental conditions that interact with each species’ genetic and metabolic background [27]. This plasticity introduces significant variability, making it difficult to extrapolate results from controlled conditions to field settings.

Some of the environmental factors that modify the phytochemical composition of plant biomass include the age of the plant and the age of the different structures, e.g., mature leaves vs. young leaves [28]; irradiance and light quality, such as UV-B and blue light [29]; temperature [27]; water availability [30]; nutrition; type of soil or substrate [25,31]; and the use of biostimulants or plant growth regulators [32]. Importantly, these factors rarely act in isolation, and their combined effects may lead to nonlinear or even contrasting metabolic responses, complicating the prediction of extract composition.

Additional environmental variables, such as elevated atmospheric CO_2_ levels, diurnal and seasonal rhythms, and the interplay of multiple stressors, can also significantly influence secondary metabolism [33,34]. For example, light serves not only as an energy source but also as a signaling cue, modulating specific biosynthetic pathways through photoreceptors and light-responsive gene networks [34,35]. Similarly, drought stress and nutrient fluctuations can trigger the differential accumulation of phenolics, flavonoids, and terpenoids, often in an organ-specific manner [36]. The effects of these factors are also modulated by the internal circadian clock in plants, which coordinates gene expression and enzymatic activity to fine-tune metabolite production across daily and seasonal cycles [33,37]. Moreover, the simultaneous occurrence of abiotic stressors, such as high temperatures and low water availability, can lead to synergistic or antagonistic interactions that reshape the phytochemical landscape of plant tissues [34]. Such interactions further increase variability among studies and highlight the need for integrative experimental designs when evaluating botanical extracts.

In addition to environmental factors affecting the composition of botanical extracts, different extraction techniques also modify the components present in the final product. The mechanism for preparing a botanical extract is as follows [22,38]: After the plant material is selected, it is rinsed thoroughly with tap water to remove surface impurities. Fresh or dry biomass—either at room temperature (~26 °C) or by freeze-drying at −80 °C—was used to safeguard bioactive compounds. The dried tissue was ground and sieved to homogenize it and maximize the surface area. Extraction success hinges on pretreatment, technique, and solvent. Physical methods, including maceration, autoclaving, and ultrasonication, are widely applied because they disrupt cell walls and increase the release of compounds. Organic solvents (methanol, ethanol, and acetone) are preferred because of their selectivity and high yields. Some studies employ anaerobic fermentation, in which fresh material is inoculated with microorganisms (e.g., manure). Microbial activity releases bioactive compounds, often enhancing the chemical and functional properties of the extract, thereby increasing its efficacy as a biostimulant. Ultimately, scalable protocols must combine efficiency, compound integrity, cost-effectiveness, and ecological safety. However, the diversity of extraction methods used across studies introduces substantial variability in extract composition, making cross-study comparisons particularly challenging.

Another variable that affects the impact of a botanical extract on plants is the method of application. Botanical extracts are applied to crops through multiple strategies tested in diverse experimental settings, including seed pretreatment (seed priming), foliar application, incorporation into nutrient solutions, amendment of substrates or soils, or combinations of these methods [22]. Differences in application methods can lead to distinct physiological responses, further complicating the interpretation of results and the identification of optimal agronomic practices.

Although the use of botanical extracts in postharvest management has been explored in various studies, further research is needed to better understand their efficacy under specific conditions, crop types, and formulation strategies. Botanical extracts are gaining recognition as sustainable alternatives to synthetic chemicals because of their ability to reduce decay, delay ripening, and preserve key physicochemical attributes in fruits and flowers [39,40]. Their bioactive constituents, including polyphenols, flavonoids, terpenoids, and essential oils, exhibit potent antimicrobial, antifungal, and antioxidant activities and have been shown to inhibit common postharvest pathogens, including *Penicillium*, *Botrytis*, *Colletotrichum*, and *Fusarium* spp. [39,41]. While these effects are consistently reported, their magnitude is rarely standardized, limiting quantitative comparisons across crops and experimental conditions.

When incorporated into edible coatings, these compounds can form semipermeable barriers that regulate gas exchange, reduce water loss, and lower respiration rates, thereby delaying senescence and improving firmness [40,42]. Additionally, when combined with other natural agents, such as chitosan, botanical extracts have synergistic effects, further increasing their ability to control microbial spoilage while maintaining sensory and nutritional quality [39]. Its efficacy has been validated in a wide range of crops, including tropical fruits such as citrus, mango, and papaya, where it helps reduce physiological weight loss and preserves antioxidant compounds [40,43,44,45]. Some extracts also trigger host defense responses, such as the induction of resistance-related pathways mediated by jasmonates or volatile aldehydes, providing an additional layer of protection against postharvest diseases [40,46]. However, discrepancies in reported effectiveness among studies suggest that these benefits are strongly influenced by formulation, concentration, and storage conditions. In addition to their role in fruit preservation, these natural treatments have shown promising applications in delicate commodities such as flowers and soft fruits, reducing microbial contamination and improving handling conditions [39,46]. Taken together, these findings highlight the potential of botanical extracts as eco-friendly tools for postharvest disease control and quality maintenance while also addressing the growing consumer demand for safer, residue-free alternatives [40]. Moreover, the variability in reported outcomes underscores the need for standardized evaluation frameworks and more systematic comparative studies.

The mechanics of obtaining a botanical extract imply that its composition is complex, potentially reflecting all the extractable and stable components found in the plant structures used as raw material. The precise profile of the extract depends on several factors, including the species and organ used as the raw material, the plant’s physiological state, preharvest and postharvest conditions, and the extraction methodology employed. This complexity, while beneficial for biological activity, complicates mechanistic interpretation and limits reproducibility across studies.

The presence of many bioactive components contributes to the remarkable biostimulating capacity of botanical extracts. Indeed, the biological effectiveness of a botanical extract is not the mere sum of its purified constituents. Mixtures of bioactive metabolites often exhibit nonlinear effects, yielding biostimulant or antimicrobial activities that surpass those of the best single molecule in standard in vitro tests. Such favorable deviations from additivity, or synergy, refer to interactions between two or more components that result in a combined effect exceeding the sum of their separate effects. Synergy occurs when extracted metabolites interact and modulate each other’s redox potential, protein binding affinity, polarity, and diffusivity [47,48]. Despite its importance, experimental evidence quantifying synergistic interactions in plant systems remains limited and represents a key area for future research.

With respect to synergy, there can be at least three complementary levels of interaction. The first is when an oxidized antioxidant can be regenerated by a companion reductant (e.g., ascorbate recycling quercetin), extending effective action and reducing intracellular pools. Second, differently substituted bioactive metabolites (e.g., phenolics) preferentially quench distinct radical species or lipid oxidation stages, thereby enabling more effective radical scavenging or metal ion chelation. Third, amphiphilic terpenes or organic acids enhance the membrane permeation or solubility of polyphenols, thereby increasing their bioavailability [49]. Although these mechanisms are conceptually well supported, quantitative evidence demonstrating their relative contributions under plant physiological conditions remains limited.

Table 1 lists some of the multiple components identified in botanical extract samples and reported in recent publications. The list includes both botanical extracts with established agricultural applications and species traditionally studied for medicinal purposes, as they may also serve as potential sources of plant biostimulants (e.g., *Moringa oleifera*). The redox impact reported for some of the components listed in Table 1 is included in one of its columns. The inclusion of species primarily studied in pharmacological contexts is intended to highlight emerging opportunities for future biostimulant development while acknowledging that the level of agronomic validation varies among species.

In addition to the above species, Godlewska et al. [118] mention the following species as potential sources of biomass and raw material for the manufacture of botanical extracts: *Aronia melanocarpa*, *Beta vulgaris*, *Equisetum arvense*, *Hippophae rhamnoides*, *Lens culinaris*, *Pteridium aquilinum*, *Polygonum aviculare*, *Pisum sativum*, *Plantago major*, and *Urtica dioica*. The wide taxonomic range of these species further highlights the diversity of potential sources but also underscores the need for systematic evaluation under comparable experimental conditions.

Table 1 highlights the wide diversity of bioactive metabolites present in botanical extracts. This chemical heterogeneity underpins the broad spectrum of biological responses observed in treated crops, ranging from the activation of antioxidant defense systems to the modulation of primary and secondary metabolism. The presence of phenolic acids (e.g., gallic acid), flavonoids (e.g., quercetin, rutin), terpenoids (e.g., limonene, carvone), and alkaloids across different botanical sources reflects a conserved ability to modulate cellular redox homeostasis, either by directly scavenging reactive oxygen species or by inducing the activity of enzymes such as SOD, CAT, and APX. Nevertheless, the magnitude of these effects is rarely reported consistently, limiting quantitative comparisons across studies.

The data in Table 1 also implicitly suggest the importance of studying even minor constituents in these extracts, which may exert significant effects through synergistic interactions with major compounds. For example, extracts from *Mentha*, *Zingiber*, and *Curcuma* contain a complex mixture of compounds whose collective impact on redox-related signaling cascades likely contributes to increased stress resistance in plants. Despite this, most studies focus on major compounds, leaving the role of minor metabolites largely unexplored.

The examples in Table 1 further demonstrate that botanical extracts appear to exert their primary physiological effects through redox-associated mechanisms, including the modulation of ROS signaling, hormone crosstalk, and the reprogramming of energy metabolism. These redox-mediated changes are often coupled with improved tolerance to abiotic and biotic stressors, effects that will be further discussed in subsequent sections. However, the relative contribution of each mechanism remains difficult to disentangle because of the multicomponent nature of these extracts.

Many extracts stimulate the activity of antioxidant enzymes, including SOD, CAT, POX, APX, GPX, and GR. Additionally, they increase the levels of nonenzymatic antioxidants and osmolytes, including ascorbate, glutathione, proline, carotenoids, and α-tocopherol. For example, garlic, cypress leaf, and liquorice root extract (0.5%; applied as a seed soaked in pea) increased enzymatic activity and mitigated oxidative damage in crops exposed to saline or heavy metals, whereas ginger and curcumin-containing extracts reduced lipid peroxidation under metal or arsenic stress. In several cases, increases in antioxidant enzyme activities are reported in the range of 20–80%, depending on the species and stress conditions, although such values vary widely among studies and experimental setups. This redox modulation often improves photosynthesis, membrane integrity, and stress tolerance, indicating that redox homeostasis is central to the biostimulant action of these extracts.

Notably, Table 1 also indicates some gaps and opportunities. While some species, such as garlic, have extensive agricultural applications, other species with well-characterized antioxidant potential—*B. officinalis*, *C. officinalis*, *E. purpurea*, and *H. perforatum*—have limited use as crop biostimulants. This highlights the opportunity to systematically evaluate medicinal and food plants as sources of botanical extracts for agriculture. This discrepancy suggests that research efforts have been unevenly distributed, with certain species being extensively studied and others remaining underexplored despite their biochemical potential.

An interesting point to consider in evaluating new botanical extracts concerns the transcriptomic response reported by [119] across different tomato genotypes (commercial and landrace) under water stress. Commercial genotypes regulate a broad range of metabolic pathways, including plant defense, indicating a response to a general metabolic disorder. On the other hand, landraces showed a more specific transcriptional response, activating drought- and stress-related metabolic pathways. The landraces presented increased expression of osmotic stress-related genes and heat stress pathways, as well as of salt transmembrane transporters and antioxidant defenses, particularly in roots. These contrasting responses highlight that plant genotype is a critical factor influencing the outcome of biostimulant application but is often overlooked in experimental designs.

Considering the above, it would be advisable for transcriptomic studies to provide information on whether a particular botanical extract induces broad changes in gene expression or whether the response is limited to specific mechanisms aimed at tolerance to a certain type of stress. Such distinctions are essential for understanding whether botanical extracts act as general stress modulators or as targeted signaling agents.

The examples in Table 1 also raise the question of biomass optimization. How can cultivation conditions be manipulated to increase the concentration and diversity of bioactive compounds in source plants to maximize the efficacy of botanical extracts? Even species cultivated for food production, not necessarily medicinal species, can be valuable sources of botanical extracts. In this case, one of the questions that should be explored is how to increase the amount (in terms of diversity and concentration) of bioactive compounds in that biomass. Addressing this question requires the integration of agronomic, physiological, and molecular approaches to optimize both biomass yield and phytochemical quality.

Recent research has demonstrated that the bioactive profile of plant biomass can be modulated through controlled environmental and agronomic interventions, as well as stress priming, thereby significantly enhancing the functional quality of the resulting extracts [15,120,121]. Controlled abiotic stresses, such as drought, salinity, and moderate temperature extremes, induce the accumulation of stress-responsive metabolites, including phenolics, flavonoids, and terpenoids [122]. However, excessive stress can negatively affect both biomass production and extract quality, suggesting an optimal stress threshold.

For example, drought stress has been shown to change the levels of bioactive metabolites in *Dendrobium moniliforme* subjected to four different drought intensities for 20 days, followed by rewatering at days 0, 5, 10, 15, and 20. The carotenoid levels peaked under severe drought conditions, whereas the total chlorophyll content increased during the early stages of drought. CAT activity decreased under stress but increased with early rewatering. Drought also increased the contents of stem polysaccharides, flavonoids, and alkaloids. PRX activity and the MDA content increase under drought conditions [123]. The authors concluded that *D. moniliforme* adapts to drought by increasing secondary metabolite production under stress and during rehydration. These results illustrate the dynamic, stage-dependent nature of metabolic responses, complicating the identification of optimal harvest or extraction timing.

On the other hand, deficit irrigation provides controlled eustress that induces secondary metabolism. Drought perception and signaling induce the production of reactive oxygen species and stress hormones, thereby upregulating pathways involved in antioxidant enzyme activity, phenylpropanoid biosynthesis, and alkaloid synthesis. Moderate stress often increases the production of bioactive compounds, whereas excessive stress negatively affects both the yield of biomass and the yield of active compounds [124]. An example is provided by [125], who applied 50% deficit irrigation to lettuce, increasing the concentrations of chicoric acid, caftaric acid, and chlorogenic acid without compromising fresh mass for commercial use. For instance, chicoric acid concentrations reached ~27,610 mg kg^−1^ DW in one cultivar, nearly doubling those observed in less responsive genotypes. Moreover, yield reductions remained cultivar dependent, ranging from negligible (~9%) to substantial (~39%) under similar irrigation regimes. These findings highlight that the benefits of deficit irrigation are strongly genotype dependent, complicating the extrapolation of results across different crop systems. Another helpful technique for inducing bioactive compounds is to prime seeds or seedlings with controlled salt shocks, usually NaCl. The perception of salinity triggers responses that modify gene expression and metabolic pathways, increasing the concentration of osmolytes and antioxidants in plants. These changes in metabolism create stress memory, enabling them to face future challenges and improve the production of bioactive compounds in biomass [120], which can subsequently be used to obtain botanical extracts. For example, faba bean plants subjected to gradual exposure to NaCl (50, 100, and 150 mM) withstand a subsequent salt shock of 200 mM NaCl while increasing the levels of bioactive compounds, such as phenolics and proline, and the expression of enzymes involved in glutathione metabolism [126]. However, the scalability of such priming strategies under field conditions remains largely untested.

In addition to field stress management, other techniques related to plant nutrition, irradiance, and spectral light balance, as well as the application of biostimulants, also improve the composition of active compounds in plant biomass.

Nutritional management also plays a pivotal role in shaping the phytochemical composition of plants. Supplementation with beneficial elements such as Se, Si, and I has demonstrated efficacy in enhancing plant resilience and promoting the synthesis of bioactive compounds. The literature provides several examples of the above. In maize under saline stress, Si application improved nearly all growth and physiological parameters. The beneficial effect was associated with the regulation of key salinity markers and with increased levels of redox metabolites, including anthocyanins, ascorbic acid, total phenols, and flavonoids [127]. However, the magnitude of these improvements is often not consistently quantified across studies, limiting direct comparisons of efficacy.

In the case of Se, the application of sodium selenate in *Origanum vulgare* under hydroponic conditions at concentrations ranging from 0.39 to 1.589 mg L^−1^ increased the concentrations of phenolic compounds, flavonoids, hydroxycinnamic acids, luteolin-7-glucoside and its derivatives, catechin, 3,4-dihydroxybenzoic acid, rosmarinic acid, and oleanolic and ursolic acids, as well as essential oils, without yield reduction [128]. These results suggest that micronutrient supplementation can increase phytochemical richness without compromising productivity, although the responses may vary depending on dose and species.

With respect to iodine nutrition, spraying lettuce in a saline environment with potassium iodate at 3 mg L^−1^ resulted in high activity of CAT, SOD, and APX, as well as high levels of ascorbate, proline, and phenolics, with increased head weight and total yield [129]. In another study on strawberry plants, the plants were grown under control or saline conditions (EC 2.5 dS m^−1^) to assess the effects of iodine on yield, antioxidants, mineral nutrition, and iodine accumulation. Under salinity, iodine increased fruit APX and CAT activities, the GSH content, and yield. In leaves, iodine increased the P, Ca, Mn, and ascorbic acid contents. Under nonsaline conditions, iodine enhances fruit phenolics and increases Ca and Mn levels [130]. Together, these findings indicate a generally positive effect of iodine on redox metabolism, although the responses differ depending on plant organ and environmental conditions.

Despite the above findings, other experiments with iodine have reported mixed results. For example, in a study of tomato plants under saline stress (100 mM NaCl) in which foliar KIO_3_ was applied every 15 days at 100 μM, iodine did not mitigate the adverse effects of salinity on fresh or dry biomass. Nevertheless, it increased fruit production by 23%. However, the levels of Ca and Mg in the fruits of plants treated with iodine, as well as GPX activity, lycopene content, and antioxidant potential, decreased [131]. This contrasting evidence highlights that the physiological and metabolic effects of iodine are highly context dependent and may involve trade-offs between yield and nutritional quality.

In other words, the combination of abiotic stresses, such as salinity, and the use of biostimulant nutrients can mitigate the impact of stress on biomass production and is associated with greater accumulation of bioactive compounds. Nevertheless, the effect may vary depending on the plant species and growing conditions. If this stress-biostimulant system is applied to biomass production to produce botanical extracts, perhaps in hydroponic, vertical farming, or other protected agricultural systems, it would be possible to obtain botanical extracts with significantly better biostimulant value.

The quality of crop biomass and its potential utility for fabricating botanical extracts can also be improved by manipulating the irradiance and spectral balance of light. PAR irradiance, through its effect on photosynthesis, is a recognized factor influencing the quality and accumulation of bioactive compounds, as well as its impact on postharvest life [132]. On the other hand, Doneva et al. [133] reported a study on *Eruca sativa*, in which two LED lighting modes, red:blue 1:1 and red:green:blue 2:1:2, were compared with conventional white light fluorescent tubes. Red: blue spectral mode increased total antioxidant activity; guaiacol peroxidase activity; and the contents of pigments, flavonoids, polyphenols, ascorbate, and polyamines. Another study with *Glehnia littoralis* confirmed that a red–blue mixture (7:5) was superior to a red–green–blue mixture and that a high proportion of blue light stimulated the accumulation of bioactive components, including the medicinally valuable compounds imperatorin, bergamottin, and coumarin [134]. These results demonstrate that light quality can be a powerful tool for modulating phytochemical composition, although optimal spectral conditions may differ among species.

Additionally, the application of biostimulants such as chitosan, salicylic acid, benzoic acid, and humic substances has been reported to increase the biosynthesis of bioactive secondary metabolites in various plant species. Biostimulants act as signaling molecules, triggering a controlled oxidative stress response that interacts with the hormonal activation of gene expression and defense-related pathways, leading to the accumulation of bioactive molecules. A recent example of the above is the response of rice to humic substances [135]. Recent studies have highlighted the effectiveness of applying chitosan, either to the soil or as a foliar spray, in increasing plant growth and stimulating the biosynthesis of secondary metabolites across various species [136]. In another example, the treatment of *Mentha piperita* with salicylic acid (150 mg/L) increased the concentration of essential oils [137]. In many cases, these increases in secondary metabolites are accompanied by increased antioxidant capacity, although the magnitude of these effects is rarely standardized across studies.

In addition to modulating the biomass composition, the extraction method significantly affects the chemical composition and functional efficacy of botanical extracts. Aqueous, hydroalcoholic, enzymatic, and supercritical CO_2_ extraction techniques differ in their ability to solubilize various classes of compounds. Aqueous extracts tend to be rich in polar compounds such as polysaccharides and some phenolics. In contrast, hydroalcoholic extracts extract a broader range of compounds, including polyphenols, flavonoids, alkaloids, tannins, and essential oils. Tailoring extraction techniques to match the desired bioactive profile is thus essential for maximizing extract potency and consistency. The topic of different extraction techniques is covered in reviews [114,138,139]. However, the lack of standardized extraction protocols across studies remains a major limitation for reproducibility and comparative analysis.

Taken together, the chemical complexity of botanical extracts provides the basis for their biological activity. Nevertheless, their effects in plants depend on how these compounds are perceived and transduced into cellular responses, as discussed in the following section. This highlights the need to move beyond compositional analyses toward a mechanistic understanding of how these multicomponent mixtures are sensed and integrated at the cellular level, linking chemical diversity with signaling and adaptive responses.

## 3. Perception, Signaling, and Putative Redox Modulation Responses After Botanical Extract Application

The biological effects of botanical extracts cannot be explained solely by their composition; rather, they depend on the perception of their components and the activation of signaling pathways that ultimately regulate plant metabolism and redox homeostasis. Botanical extracts have long been recognized as complex mixtures of bioactive compounds that can modulate cellular homeostasis. In addition to their direct antioxidant properties, they can activate or inhibit signaling cascades that regulate antioxidant defenses, inflammatory responses, and mitochondrial function [140,141]. The molecular perception of these compounds often involves redox-sensitive proteins, receptor-mediated recognition, and the modulation of pathways such as PI3K/Akt, NF-κB, Nrf2, AMPK–SIRT1, and MAPK, which ultimately converge on redox-modulation responses. Although these signaling pathways have been extensively characterized in animal systems, their inclusion here is intended to illustrate general redox-sensitive regulatory principles, as functionally analogous mechanisms based on ROS signaling and redox regulation are well established in plant systems. In plants, these responses are primarily mediated through reactive oxygen species (ROS) signaling, calcium flux, mitogen-activated protein kinase (MAPK) cascades, and phytohormone-regulated pathways (e.g., those involving salicylic acid, jasmonate, and abscisic acid), which collectively coordinate stress responses and metabolic reprogramming.

The effects of botanical extracts on metabolic and physiological behavior, as well as gene expression, do not depend on a single component but rather on the synergistic influence of all present components. In addition to the bioactive compounds listed in Table 1, botanical extracts contain inorganic nutrients such as Fe and Zn, as well as other components, including amino acids and peptides, possibly low-molecular-weight proteins, oligomers, and polysaccharides, and components subject to varying levels of degradation that qualify as DAMPs. All components of the original plant biomass are extracted with varying efficiencies depending on the extraction method used [22].

This section describes the putative perception and response process following the application of multiple components in a botanical extract, with emphasis on redox responses. In plants, redox-sensitive signaling pathways involving ROS, antioxidant enzymes, and redox-dependent transcription factors are increasingly recognized as key mediators of responses to botanical extracts, collectively determining adaptive outcomes. By analogy, in human and animal systems, these mechanisms have been more extensively characterized: electrophilic phytochemicals can modify cysteine residues on redox-sensitive proteins, such as Keap1, thereby promoting Nrf2 nuclear translocation and upregulating antioxidant response element (ARE)-driven gene expression [142,143]. Moreover, botanical extracts can also modulate NF-κB and AP-1 activity [144].

The above biomedical findings are cited here solely to illustrate conserved redox-regulatory principles, not to imply equivalent mechanisms in crops. These biomedical findings are cited here solely to illustrate conserved redox-regulatory principles, not to imply equivalent mechanisms in crops. Notably, throughout this section and the review, the evidence cited varies in its level of attribution. Some responses have been demonstrated using whole botanical extracts applied under agronomic conditions. In contrast, others derive from studies using isolated or purified compounds or from general oxidative stress frameworks that are not specific to botanical extracts. Where this distinction is relevant to mechanistic interpretation, it is noted explicitly.

**Figure 2:**
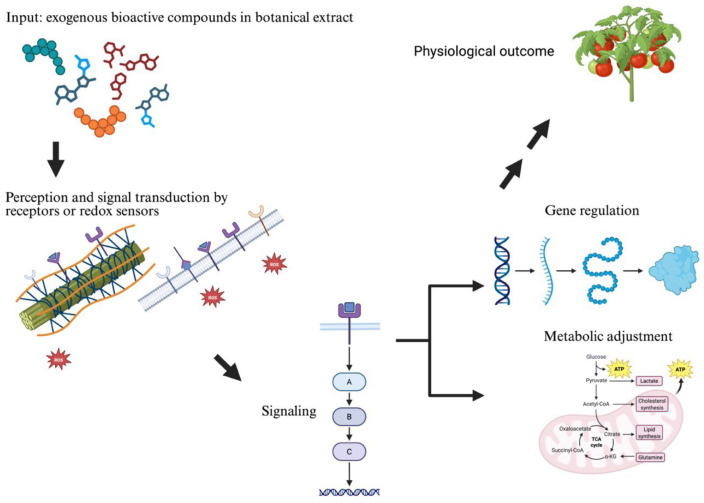
Sequence of events related to biostimulation with botanical extracts.

Like other biostimulants, the reaction of plants to the application of a botanical extract involves a sequence of events that begins with the (1) perception of the extract’s components, after which the cellular receptors (2) transduce the information in the form of cellular (3) signaling that triggers the action of second messengers (e.g., ROS, Ca^2+^, and MAP kinases) that (4) modify enzyme activity, metabolism and gene expression [145] (Figure 2). This sequential framework provides a conceptual link between extract composition and the downstream physiological responses observed in plants.

The initial interaction between exogenous molecules and the plant (perception) occurs at the cuticle, epidermis, or seed coat. Plant surfaces contain structural and biochemical receptors that sense and transduce changes in osmotic pressure, pH, and redox potential, as well as the presence of specific bioactive compounds [146]. Receptor proteins primarily perceive many developmental, biotic, and abiotic signals at the cell wall and plasma membrane. However, crosstalk between signaling pathways is triggered by extracellular and intracellular receptors [147]. At the cell surface, receptor-mediated recognition also plays an essential role. In plants, equivalent outcomes are achieved through ROS bursts, calcium signaling, and MAPK cascades, which together coordinate early adaptive responses to botanical extract components. In a comparative context, in human and animal systems, botanical extracts have been shown to modulate receptor tyrosine kinases and the PI3K/Akt signaling axis, which are linked to mitochondrial function and NADPH oxidase (NOX) activity, leading to improved mitochondrial integrity and reduced ROS production [141,148]; however, direct evidence for analogous receptor–kinase cascades activated by botanical extracts in crop plants remains limited.

During the subsequent signaling phase, ion flux, especially Ca^2+^ influx into the cytoplasm, represents one of the earliest measurable responses, establishing a chemical signature that varies depending on the identity, concentration, and temporal profile of the stimulus [149]. In parallel, apoplastic and symplastic oxidative bursts occur because of the activation of respiratory burst oxidases, leading to the transient accumulation of ROS with redox signaling functions [150]. These early signaling events represent key mechanisms through which the perception of botanical extracts is translated into downstream physiological responses in plant systems.

Another possible signaling mechanism of botanical extracts may involve gasotransmitters, such as NO and H_2_S. In the biomedical field, botanical extracts are recognized as natural sources of gasotransmitters [151,152,153], and their components can act as inducers [154,155]. However, to our knowledge, there is currently no direct experimental evidence linking the application of botanical extracts to gasotransmitter-mediated signaling in plant systems. Therefore, this possibility should be considered a working hypothesis rather than an established mechanism. Nevertheless, in plants, signaling molecules such as NO are well-established regulators of redox homeostasis and stress responses, suggesting that future research should explore whether botanical extracts can modulate these pathways under agronomic conditions. The signaling induced by botanical extracts, which in some ways resembles the oxidative stress stimuli caused by abiotic stress, may result in (1) posttranslational modifications that modulate metabolism while also causing changes in gene expression through alterations in (2) transcriptional regulation and (3) the cellular epigenome. These adjustments are highly important for plant adaptive responses and development [156,157,158]. Thus, botanical extracts may act as controlled redox stimuli, triggering adaptive responses rather than causing detrimental oxidative damage.

The ROS produced upon contact with botanical extracts act as secondary messengers, propagating signals within the cell and to neighboring cells and inducing posttranslational modifications. ROS bursts modify the redox state of proteins, thereby modulating the activity of redox-sensitive proteins, such as transcription factors, MAPKs, calcium-dependent protein kinases, and phosphatases [159]. These redox-based posttranslational modifications serve as a biochemical imprint, linking early perception to downstream signaling cascades and thereby orchestrating signal amplification and specificity through the phosphorylation of transcription factors and other regulatory proteins [147,160]. This ROS-mediated signaling framework serves as a central hub connecting perception to metabolic reprogramming in plants.

Additionally, redox signals can lead to broad posttranslational modifications, including the oxidation of cysteine thiols, S-glutathionylation, S-nitrosylation, and protein carbonylation. These modifications dynamically alter enzyme activities and protein–protein interactions in pathways associated with photosynthesis, respiration, and nutrient assimilation [8]. However, despite the importance of these adjustments for crop metabolism, information regarding the impact of botanical extracts is scarce or absent. This gap highlights the need for future studies integrating redox proteomics to better understand the molecular targets of botanical extracts in plant systems.

Additionally, botanical extracts may contain ABA, auxins, salicylic acid, and other hormones that work synergistically to influence stress-responsive metabolic pathways via integrated networks of hormone crosstalk, gene regulation, and physiological responses [161]. Such hormonal interactions further reinforce the integrative nature of botanical extract-induced responses, linking redox signaling with growth and defense regulation.

The response phase following the application of botanical extracts includes the transcriptional reprogramming of genes involved in antioxidant defense, redox modulation, secondary metabolism, and hormone signaling. In a recent study, aqueous extracts of different plant species (*Trifolium pratense*, *Pisum sativum*, *Solidago gigantea*, and *Hypericum perforatum*, among others), applied as a foliar spray at 0.5% to cabbage (*Brassica oleracea*) seedlings, were shown to regulate genes associated with defense and redox regulation [162]. These findings support the concept that botanical extracts induce targeted transcriptional adjustments rather than nonspecific stress responses.

In another example, soaking pea (*P. sativum*) in liquorice root extract (0.5%; 5 g L^−1^) for 2 h increased the activity of ascorbate, glutathione, and the antioxidant enzymes CAT, SOD, APX, and GR under 150 mM NaCl salinity stress [163].

In a study linking nanomaterials with botanical extracts, a supercritical CO_2_ extract of garlic (*Allium sativum*) encapsulated in nanoscale liposomes was applied at 240 g ha^−1^ via foliar spraying to wheat (*Triticum aestivum*) at the BBCH12 growth stage under greenhouse conditions. The results suggested increased expression of ABA pathway genes and pathogenesis-related (PR) genes [164]. Together, these examples illustrate how botanical extracts can modulate both antioxidant systems and hormone-regulated defense pathways in plants. However, it is worth noting that both studies were conducted under controlled greenhouse or laboratory conditions; field-scale validation of these transcriptional responses remains limited, and their reproducibility under variable agronomic conditions has yet to be established.

Two additional regulatory layers, small RNA signaling and epigenetic modification, represent speculative but potentially relevant mechanisms through which botanical extracts could influence plant adaptive responses and are noted here as priorities for future research rather than established pathways. With respect to small RNAs, sRNAs such as miRNAs and siRNAs are well-established regulators of posttranscriptional gene expression in plants [165], and miRNAs are known to move systemically within and between organisms [166]; however, there is currently no direct evidence that the miRNAs present in botanical extracts modulate gene expression in recipient plants.

Similarly, while epigenetic modifications induced by botanical extracts have been characterized in the biomedical field [167,168,169], their role in crop systems remains, to the best of the authors’ knowledge, unexplored. Both mechanisms, if experimentally confirmed in agricultural contexts, would substantially expand the mechanistic framework for understanding long-term biostimulant effects.

The integrated set of perception and signaling events triggered by botanical extracts leads to adaptive redox responses in crops, including increased mitochondrial bioenergetics and the modulation of antioxidant pathways. These effects help regulate plant metabolism and improve tolerance to abiotic stress, thereby supporting crop resilience under adverse environmental conditions. However, challenges remain in optimizing the bioavailability of these compounds in the field, distinguishing between synergistic and antagonistic effects among extract components, and determining accurate dosing to maximize their metabolic and redox impact in agricultural applications [170]. Overall, this integrative framework highlights the central role of redox signaling as a unifying mechanism underlying the biostimulant action of botanical extracts.

## 4. Reprogramming of Metabolism and Gene Expression by Botanical Extracts

The signaling events triggered by botanical extracts ultimately converge into metabolic and transcriptional reprogramming, which determines the final physiological outcome in plants. However, the extent and direction of these responses are highly dependent on factors such as extract composition, plant species, developmental stage, and environmental conditions, which introduce variability across studies and complicate direct comparisons. The application of botanical extracts via foliar or fruit spraying, seed priming, or soil or nutritive solution root treatment triggers a complex cascade of cellular events, leading to morphological, physiological, and molecular adjustments. These responses align with the recognized capacity of plants to integrate environmental information through coordinated signaling and adaptive responses [171,172]. In this context, botanical extracts can serve as valuable sources of information, providing environmental signals that reprogram plant metabolism and gene expression [158]. These compounds act as potent biostimulants that reprogram plant metabolic pathways and gene expression through a complex array of bioactive compounds, such as phytohormones, polysaccharides, vitamins, amino acids, and secondary metabolites, that synergistically trigger intricate signaling cascades, leading to improved nutrient uptake, enhanced growth, and increased stress resistance [173,174]. Nevertheless, the relative contribution of individual compound classes versus synergistic interactions remains largely unresolved, representing a key limitation in the current mechanistic understanding.

In general, adaptive reprogramming refers to the dynamic changes that cells, tissues, or complete organisms undergo in response to internal or external stimuli to maintain function, improve survival, or enhance fitness. It involves modifying gene expression, metabolism, signaling pathways, or epigenetic states to adapt to new conditions [175]. In adaptive reprogramming, metabolic reprogramming refers to the controlled alteration of cellular metabolic pathways in response to developmental cues, environmental changes, or stress conditions. While this concept is well established, its quantitative characterization of plant responses to botanical extracts remains limited, with most studies reporting qualitative rather than quantitative shifts in metabolic activity.

For example, *Ascophyllum nodosum* seaweed extract applied to rapeseed (*Brassica napus*) at a foliar concentration of ≤0.05% *v*/*v*, which has been reported to be optimal for eliciting plant responses, upregulated the expression of nutrient transporter genes such as *BnNRT1.1*, *BnNRT2.1*, *BnSultr4.1*, and *BnSultr4.2*, thereby increasing nitrogen, sulfur, and iron uptake and assimilation [174].

In several cases, these improvements translate to measurable increases in plant growth and yield-related parameters, often reported at 10–30% depending on the species and experimental conditions. However, these effects are not consistently observed across all studies. Indeed, the magnitude of these responses is strongly influenced by extract composition and application conditions, and the lack of standardization among studies limits direct quantitative comparison and mechanistic interpretation [174].

Similarly, the application of an alfalfa protein hydrolysate (0.1–1 mL L^−1^) resulted in increased biomass, chlorophyll content, and soluble sugar content in tomato plants. For instance, shoot biomass increased by up to 37% and root biomass by 21% under optimal application rates, indicating a clear physiological impact of the treatment. The observed favorable effect was associated with alterations in the expression of genes encoding proteins that impact various metabolic pathways. Of note for this review are those proteins related to metabolism or redox homeostasis whose abundance was significantly modified in response to biostimulant application, such as cytochrome 450, glutathione-S-transferase, lactoylglutathione lyase, alternative oxidase 1A, and antioxidant-related genes such as APX, CAT, thioredoxins, hemoglobins, glutaredoxins, dehydroascorbate reductase, CTF2A monooxygenases, and ferredoxin [176].

At the transcriptomic level, the above response involved the differential regulation of more than 1000 genes per organ, with fold-change thresholds ≥ 2 and, in some cases, extreme upregulation exceeding 100-fold, highlighting the intensity and breadth of the metabolic reprogramming induced by the extract. Nevertheless, these results were obtained under controlled conditions with a single extract formulation and crop species; whether comparable transcriptomic responses occur under field conditions, where environmental variability and differences of extraction are common, remains unknown.

Metabolic reprogramming enables cells and organisms to optimize the production and utilization of energy, photosynthates, and other molecules, as well as redox equivalents, to support specific physiological states [177,178]. In the context of botanical extracts, this optimization is often linked to enhanced stress tolerance, although the trade-off between growth and defense has not been consistently evaluated across studies.

Metabolic reprogramming occurs within the context of a developmental program, a dynamic and coordinated set of metabolic, physiological, and gene expression processes that guide the transition of plant cells and tissues from an initial state at time t0 to a specific functional state at time t1 (t1 > t0). This transition occurs in response to endogenous and exogenous physical or chemical signals [179]. In parallel, botanical extracts modulate secondary metabolism, often activating the phenylpropanoid pathway and increasing the production of flavonoids, phenolic acids, and carotenoids, which are key metabolites that facilitate defense and stress tolerance [173,180]. Despite these consistent trends, the magnitude of metabolite accumulation varies widely across species and environmental contexts, highlighting the need for standardized experimental frameworks.

Plants perceive exogenous molecules in the botanical extract via membrane-bound receptors, kinases, and redox sensors, which induce metabolic reprogramming [181]. These events initiate signal transduction cascades involving Ca^2+^ flux, ROS waves, mitogen-activated protein kinase (MAPK) activation, and hormone crosstalk. The results indicate that botanical extracts may mimic endogenous signals, such as those derived from stress hormone action and signaling, including ethylene, auxin response modulation, and the MAPK signaling pathway, thereby inducing stress-analogous response networks with a relevant redox component [182,183]. Additionally, botanical extracts are likely to induce heritable epigenetic changes, resulting in long-term transcriptional stress memory or biostimulation [183]. However, direct experimental evidence supporting long-term epigenetic effects in crops remains scarce, and most conclusions are inferred from short-term studies, representing an important gap for future research.

Hormonal regulation is central to this process; for example, *Ascophyllum nodosum* extracts can transiently increase cytokinin levels and activate cytokinin-responsive genes (e.g., ARR5) while suppressing auxin signaling, thereby modifying root architecture and contributing to a strategic rebalancing of the growth–defense trade-off [184,185]. However, the magnitude and duration of these hormonal effects vary considerably across studies, depending on the extract formulation, application method, and plant species, limiting the generalizability of these responses.

As with other environmental stress stimuli, the metabolic reprogramming response to a botanical extract is accompanied by a redirection of photosynthates toward defense. This growth–defense trade-off is an evolutionarily conserved strategy that enables plants to respond adaptively to environmental challenges [186]. Nevertheless, not all studies report a clear trade-off, and in some cases, simultaneous improvements in growth and defense have been observed, suggesting that the balance between these processes is strongly context dependent.

This trade-off can result from the fact that many compounds in botanical extracts originate from metabolic responses to biotic or abiotic stress in the source plant [187]. These molecules thus carry chemical signatures of ecological history or experiences; they may be considered analogous to allelochemicals that communicate information to elicit a response [188]. In addition, botanical extracts act as elicitors, priming plants through the upregulation of antioxidant enzymes (SOD, CAT, and APX) and activating the salicylic acid (SA) and methyl jasmonate (MeJA) pathways via transcription factors such as WRKY, MYB, and AP2/ERF, which are often coupled with MAPK cascades that increase phytoalexin production [173,180,189]. While these responses are widely reported, the degree of enzyme induction and pathway activation varies substantially across studies, and quantitative comparisons are often limited by differences in experimental design and reporting metrics.

Like other biostimulants, the composition, dose, application timing, and environmental conditions of a botanical extract can influence the growth–defense trade-off. Low concentrations of extracts (equivalent to eustress) may act as priming agents, preparing plants to activate defenses more rapidly in response to future stress without significant growth penalties. In contrast, higher concentrations or frequent applications (equivalent to distress) may push the metabolic balance more strongly toward defense, impairing biomass accumulation [190]. This dose-dependent response highlights a narrow optimal window of application, which is rarely standardized across studies and represents a major limitation for practical agricultural implementation. Multiomic analyses, combining transcriptomics, proteomics, and metabolomics, have revealed the modulation of enzymes such as oxidosqualene cyclase, β-amyrin synthase, and UDP-glycosyltransferases, redirecting metabolic flux toward high-value compounds. In *Panax ginseng*, MeJA treatment upregulated PgMYB2, increasing dammarenediol synthase expression and enhancing ginsenoside biosynthesis [180,191]. However, it should be noted that MeJA here acts as an isolated elicitor, not as a component of a whole botanical extract. Specifically, elicitation strategies have been reported to significantly increase ginsenoside accumulation, with some studies showing several-fold increases in key intermediates and associated biosynthetic enzymes, highlighting the strong responsiveness of the pathway to jasmonate signaling.

Importantly, the abovementioned trade-off is not necessarily detrimental. In modern agroecosystems, where abiotic stress and pathogen pressure are common, prioritizing temporary defenses may increase overall plant fitness and yield stability. Thus, botanical extracts can serve as both elicitors and modulators of plant phenotypic plasticity, fine-tuning the balance between defense and development in an ecologically relevant manner [187]. Translating this potential into consistent agronomic practices, however, requires standardized application protocols and field trials across diverse crop systems and environmental conditions, constituting a critical gap in the current literature. Furthermore, in isolated human cells, botanical extracts exhibit adaptogenic properties, inducing heat shock proteins (Hsp70) and stress-responsive transcription factors (e.g., HSF1), thereby expanding the homeostatic range of cellular functions and improving resistance against environmental stressors [192]. Although these adaptogenic responses are well documented in animal systems, their relevance here lies in illustrating general stress-response principles. In contrast, in plants, comparable effects are mediated through ROS signaling, antioxidant systems, and hormone-regulated pathways.

**Figure 3:**
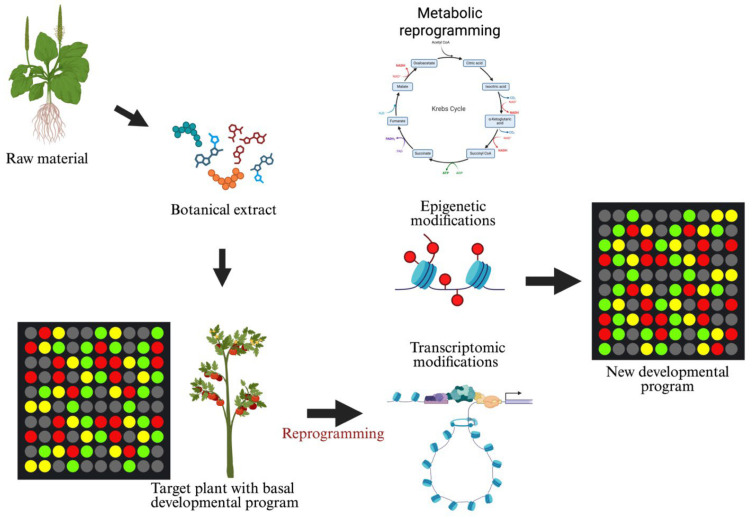
Schematic diagram of adaptive reprogramming that occurs after the application of a botanical extract. Metabolic reprogramming is part of adaptive reprogramming.

As with previous topics, metabolic and gene expression reprogramming have been studied most extensively in the biomedical field [193,194]. With respect to the agricultural use of botanical extracts, although molecular response mechanisms analogous to those in mammalian cells might be expected, little information is available. This imbalance in available evidence highlights a critical knowledge gap, particularly in the translation of mechanistic insights into agronomically relevant outcomes under field conditions. A schematic model of adaptive reprogramming, which includes metabolic reprogramming, is shown in Figure 3.

Considering the average composition of plant extracts (Table 1) and the impact of their components on plant redox homeostasis, which components of plant extracts should be balanced, increased, or decreased to improve their potential as crop biostimulants? Addressing this question requires not only compositional analysis but also quantitative evaluation of dose–response relationships and redox dynamics in target crops. Assuming that a substantial part of the beneficial impact of a botanical extract is related to redox modulation in the target crop, those components that promote a rapid, but not sustained, redox response (e.g., nonprotein antioxidants, antioxidant protein cofactors, and nonpolymeric pro-oxidants) are expected to produce an oxidative burst triggering defense responses without maintaining prolonged production of reactive species. Ascorbate, glutathione, phenolic and flavonoid compounds, peptides, oligosaccharides, and nutrients such as Zn and Se may improve the quality of the botanical extract. On the other hand, the presence of transition metals relevant to Fenton reactions and the induction of ROS by phenolics, such as Fe and Cu, as well as the presence of alkaloids at high concentrations, should be avoided. However, the optimal balance between pro-oxidant and antioxidant components remains poorly defined and is likely to vary across crop species and environmental conditions.

To increase the concentration of the abovementioned beneficial compounds, simple agronomic biostimulation or biofortification techniques can be used, either with conventional nutrients or with nanonutrients [195]. In many cases, the responses of treated plants match the above findings: increased levels of ascorbate, glutathione, phenolic, and flavonoid compounds, as well as increased concentrations of trace nutrients such as Zn and Se. Nevertheless, the magnitude of these increases is inconsistently reported, and standardized quantitative benchmarks remain lacking.

While conventional genetic improvement techniques or those involving genetic transformation or editing can achieve substantial improvements in the yield of bioactive compounds in medicinal species, it should not be overlooked that agronomic management, through nutrition, biostimulation, irrigation management, temperature, irradiance, and spectral balance, can also improve biomass quality in terms of biostimulant potential. The medicinal plant *H. perforatum* is an example of a technique used to strengthen the phytochemical composition through agronomic management strategies, as described previously [196,197]. However, the transferability of these findings to other species remains uncertain because of species-specific metabolic regulation.

Another point worth highlighting is that the previously mentioned agronomic techniques can be applied to improve the biomass of the species used for food production. Most likely, unused plant biomass and postharvest residues from biostimulated plants (e.g., corn, wheat, and broccoli), among others, can be used to produce plant extracts with significant biostimulant potential. If possible, this would be a way to add value to these residues. However, experimental validation of this circular approach is still limited and requires systematic evaluation.

## 5. Conclusions and Future Directions

The literature suggests that botanical extracts can induce controlled ROS signaling, which primes plants for increased tolerance to abiotic and biotic stressors. The response is associated with altered expression of genes encoding antioxidant enzymes, upregulation of nonenzymatic antioxidant pathways, and modulation of stress hormone crosstalk. Transcriptomic and proteomic analyses revealed that the extracts modulated stress-responsive pathways, the synthesis of bioactive metabolites, and signaling cascades that modify the cellular redox state, in addition to potentially affecting the proteome, metabolome, and ionome of the target plants.

Despite significant advances, several knowledge gaps remain. For example, as with other biostimulants, the variability in plant responses to botanical extracts is substantial. This variability is partly attributable to differences in extract composition, application methods, crop species, developmental stages, and the growth environments of the target crops. When research advances regarding the use of extracts in practice are translated into practice, this can affect the feasibility of knowledge transfer.

Crops biofortified with Fe, Zn, Se, I, and Si can be potential sources of biomass for the production of high-quality botanical extracts. To date, the focus of biofortification and biostimulation has been on obtaining edible organs with high nutritional and nutraceutical qualities. Nevertheless, biomass not utilized as food is likely also enriched with bioactive metabolites. The same argument applies to crops biostimulated with amino acids, salicylic acid, chitosan, or humic substances, among others. The practical objective is to improve the yield and nutraceutical quality of the organs consumed. Nevertheless, there is also the opportunity to obtain useful biomass for the production of botanical extracts.

Furthermore, the complexity of the extract composition poses challenges for mechanistic elucidation and commercial standardization. Currently, both points vary between species and growing conditions. A deeper understanding of the composition and the factors that modify it, in pursuit of greater control and the subsequent standardization of botanical extracts, is a current challenge. A detailed metabolomic characterization of botanical extracts will be particularly relevant for identifying the specific compounds and synergistic combinations responsible for redox modulation. Furthermore, combining metabolomic and multiomic information with artificial intelligence-based optimization techniques could facilitate the design of predictive schemes for specific extraction applications across crop genotypes and agroclimatic zones.

Future research should prioritize field-scale validation under variable environmental conditions to bridge the gap between controlled-environment studies and real-world agricultural systems. This includes evaluating long-term impacts on crop yield stability, quality traits, and soil health, as well as understanding interactions with the plant microbiome. The integration of microbiome profiling with plant physiological and biochemical response data could reveal additional indirect benefits of botanical extracts, such as enhanced nutrient cycling and biological pest suppression.

Relatedly, the combined use of nanomaterials and botanical extracts can contribute to a hybrid biostimulation system. Encapsulation, nanoformulations, and controlled-release systems can increase the stability and bioavailability of redox-active compounds in botanical extracts, thereby improving field-level efficacy and scalability. Moreover, developing cost-effective, eco-friendly nanodelivery systems is crucial for ensuring the adoption of these technologies in smallholder and resource-limited farming systems.

Ultimately, interdisciplinary collaboration among plant scientists, agronomists, microbiologists, and data scientists will be crucial for accelerating the translation of laboratory findings into practical, scalable agricultural solutions. Establishing standardized guidelines for extract preparation, quality control, and efficacy testing will not only improve reproducibility but also facilitate regulatory approval and market acceptance, positioning botanical extracts as cornerstones of next-generation sustainable agriculture.

## Figures and Tables

**Table 1:** List of different botanical extracts, some of the reported components, and the redox impact of the latter.

Botanical Extract	Representative Chemical Components	Representative Redox Impact of Chemical Component(s) in Planta
*Allium sativum* bulb	Alliin, diallyl sulfide, ajoene, carvacrol, geraniol, quercetin, apigenin, rutin [50]	The aqueous garlic extract enhances the activity of antioxidant enzymes, including SOD and PRX, in tomatoes and eggplant [51,52].
*Aloe vera* inner-leaf gel	Quercetin, kaempherol, aloesin, rutin, caffeic acid, cinnamic acid, vanillic acid, and acemannan [53]	Application of 60 mL L^−1^ of *Aloe vera* extract enhanced the growth and productivity of *Silybum marianum*. In contrast, a concentration of 40 mL L^−1^ *A. vera* extract increased both silybin content and chalcone synthase gene expression [54].
*Artemisia vulgaris* leaves	Artemisinic acid, rutin, luteolin, kaempferol-3-*O*-glucoside, tracheloside [55], hydroxycinnamic acids (caffeic, sinapic, p/m-coumaric, ferulic, homovanillic and chlorogenic) and hydroxybenzoic acids (p-hydroxybenzoic, gallic, syringic, salicylic and gentisic) [56].	*Artemisia vulgaris* extract increased chlorophyll, carotenoids, proline, and polyphenol levels in potato plants [57]. *A. vulgaris* extract (leaves and stems) improved germination and seedling growth in some plant species, while it showed no impact in others [58].
*Azadirachta indica* (neem) seeds	Azadirachtin, astaxanthin, cinobufagin, anodendroside, marinobufagin [59]	Azadirachtin (2.4% azadirachtin A) applied to leaves triggers a defense response in tomatoes comparable to that elicited by *Bacillus subtilis* via Induced Systemic Resistance (ISR). Foliar treatment with *B. subtilis* activated ISR via the jasmonic acid signaling pathway and promoted the synthesis of secondary metabolites, including flavonoids, phytoalexins, and auxins. Alterations in sterol and terpene profiles, along with elevated glucosinolate levels, were also detected [60].
*Borago officinalis* flowers	Rosmarinic acid, astragalin, rutin, linoleic acid [61]	Primarily studied for pharmacological applications; antioxidant properties documented in vitro. Agronomic validation in plant systems remains limited [62,63].
*Brassica juncea* seeds	Glucosinolates: 4-hydroxyglucobrassicin, glucobarbarin, glucobrassicanapin, glucoerucin, gluconapin, gluconasturtiin, and neoglucobrassicin [64]	Allyl isothiocyanate (AITC) is a phytochemical associated with plant defense in plants from the Brassicaceae family. AITC has long been recognized as a countermeasure against external threats and is also involved in initiating defense-related mechanisms, such as stomatal aperture regulation. At the level of redox modulation, AITC induces depletion of glutathione and the upregulation of glutathione S-transferases in *Arabidopsis thaliana* [65].
*Calendula officinalis* flowers	Lupeol, erythrodiol, calenduloside, rutin, narcissin, esculetin, cubenol, limonene, calenduloside B [66]	*C. officinalis* exhibits strong ROS scavenging activity and modulation of glutathione-related enzymes [67], while phenolic-rich extracts mitigate oxidative stress and restore antioxidant enzyme balance in planta [68].
*Camellia sinensis* leaves (green-tea extract)	Catechins, caffeine, theanine, gallic acid [69]	Exogenous application of catechin enhances photosynthesis, plant growth, leaf expansion, antioxidant defense mechanisms, reactive oxygen species signaling, redox balance, and hormone metabolism under environmental stress conditions [70].
*Citrus sinensis* peel	Naringin, rutin, hesperidin, melittoside [71]	The physiological and biochemical effects of hesperidin (100 μM) and chlorogenic acid (50 μM) were evaluated in *Zea mays* under arsenate stress (100 μM). Hesperidin and chlorogenic acid enhanced the activities of SOD, CAT, PRX, glutathione S-transferase, and glutathione peroxidase under stress, effectively reducing H_2_O_2_ accumulation and lipid peroxidation [72].
*Cymbopogon citratus* leaf extract	Luteolin, apigenin, di-C-glycosylflavones, tannins [73]	*C. citratus* extracts possess antioxidant properties, enabling their use in food preservation, as herbicides and insecticides, and offering a broad range of medicinal uses [74]. *C. citratus* extract enhances germination, growth, and antioxidant metabolism (phenolics, enzyme activity) in wheat, indicating a biostimulant redox effect [75]
*Echinacea purpurea* leaves, flowers, roots	Mannitol, benzoic acid, betulin, campesterol, β-sitosterol [76]	*Echinacea purpurea* root extracts possess antioxidant properties, enabling their use in food preservation and offering broad therapeutic potential [77]. Extracts from *E. purpurea* (leaves and flowers) showed limited biostimulant activity, with minimal effects on shoot and root development in cabbage seedlings; its use as a plant biostimulant appears to be limited [78].
*Cupressus macrocarpa* leaf extract	Secondary metabolites, phenolics, flavonoids, saponins, tannins, terpenes, and essential oils	Leaf extract applied to salt-stressed zucchini enhanced SOD, CAT, APX, GPX, GR, and DHAR activities; increased ascorbate, glutathione, and proline; upregulated antioxidant genes (CuZnSOD2, CAT1, APX, GR, DHAR, PrxQ); and outperformed salicylic acid under both saline and nonsaline conditions [79].
*Curcuma longa* rhizome	Curcumin and derivatives, calebin A, gallic acid, rutin [80]	Purified curcumin (1–20 μM) reduced oxidative stress markers (H_2_O_2_, MDA), enhanced glutathione redox cycle activity, increased glucosinolate and phenolic accumulation, and promoted ROS–phytohormone crosstalk in arsenic-stressed spinach [81]. Note: this study used purified curcumin, not a whole C. longa extract.
*Foeniculum vulgare* and *Ammi visnaga* seed extract	Osmoprotectants, antioxidants, and trace nutrients	The seed extracts—rich in macro- and micronutrients, α-tocopherol, phenolics, and glutathione GSH—strengthened salt-stressed cowpea plants’ antioxidant defenses by modulating osmoprotectants, such as proline and soluble sugars, and enhancing CAT, PRX, APX, and SOD enzymes and nonenzymatic systems, including carotenoid and glutathione levels [82].
*Glycyrrhiza glabra* root	Glycyrrhizin, glycyrrhizic acid, isoliquiritigenin, licochalcone A, 18-β-glycyrrhetinic acid, glabrene [83]	Field application of licorice root extract (0.5%; seed priming and/or foliar spray) to salt-stressed Phaseolus vulgaris enhanced antioxidant enzyme activity, reduced MDA, H_2_O_2_, and Na^+^ levels, and improved growth, yield, K^+^/Na^+^ ratio, and membrane stability [84]. Two field trials assessed the effects of licorice root extract (0.5%; 5 g L^−1^ in distilled water), applied as seed priming and/or foliar spray, on
*Hypericum perforatum* leaves and flowers	Hyperforin, adhyperforin hyperoside, rutin, isoquercitrin, quercitrin [83]	Primarily studied for pharmacological applications; antioxidant properties documented in vitro. Agronomic validation in plant systems remains limited [85,86].
*Medicago sativa* leaves	Chlorogenic acid, rutin, quercetin, kaempferol, genistein, vitexin [87]	Primarily studied for pharmacological applications; antioxidant properties documented in vitro. Agronomic validation in plant systems remains limited [88,89,90].
*Moringa oleifera* leaves	Phenolic acids (chlorogenic, caffeic), fatty acids, amino acids, flavonoids (rutin, quercetin, kaempferol), glucosinolates, tocopherols [91]	Under oxidative stress, exogenous chlorogenic acid application to apple leaves mitigated chlorophyll loss, decreased photosystem II efficiency, reduced membrane damage and lipid oxidation, and enhanced antioxidant enzyme activity. Phenolic concentrations significantly increased, and the expression of genes involved in antioxidant defense was modulated [92].
*Olea europaea* leaves	Homogentisic acid, hydroxybenzoic acids, caffeic acid, vicenin, and luteolin [93]	*Olea europaea* leaf extracts possess antioxidant properties, enabling their use in food preservation and offering a broad therapeutic potential [94,95,96]. Its use as a plant biostimulant appears to be limited.
*Punica granatum* peel	Punicalagin, rutin, ellagic acid, gallic acid, and anthocyanins [97]	Primarily studied for pharmacological applications; antioxidant properties documented in vitro. Agronomic validation in plant systems remains limited [98,99].
*Rosmarinus officinalis* leaves	Carnosic acid, rosmarinic acid, luteolin, apigenin, caffeic acid [100]	The study measured endogenous levels of carnosic acid and α-tocopherol—lipophilic antioxidants in the chloroplasts of *Salvia officinalis* during a drought-recovery cycle. Drought significantly reduced salvia leaf water content, and as stress intensified, α-tocopherol and carnosic acid levels declined while the oxidation products, rosmanol and isorosmanol, increased. Carnosic acid serves a similar antioxidative role in both rosemary and salvia, emphasizing that drought resistance relies on the combined action of multiple antioxidants rather than a single mechanism [101].
*Salix alba* bark	Salicin, chlorogenic acid, rutin, epicatechin [102]	Application of *Salix alba* root powder and *Bacillus thuringiensis* to wheat significantly increased shoot dry weight, root fresh weight, and CAT and APX levels. The combined use of *B. thuringiensis* and *S. alba* root powder promoted plant growth and defense responses under elevated soil cadmium concentrations [103].
*Solidago gigantea*, *S. canadensis*, *S. virgaurea*, *S. graminifolia*, *S. speciosa* leaves and flowers.	Chlorogenic acid, rutin, hyperoside, quercitrin, isoquercitrin [104]	*Solidago gigantea* (leaf extract) exhibited biostimulant potential, contributing to improved seedling growth (shoot/root development and biomass) and enhanced photosynthetic pigment content in cabbage, with effects dependent on extract concentration and associated with modulation of antioxidant-related metabolism [105,106].
*Silybum marianum* seeds	Silybin A, silydianin, taxifolin, quercetin, sylimarin [107]	*Silybum marianum* seed extracts possess antioxidant properties and have a range of uses in humans and plants [108]. However, its use as a botanical extract in agriculture appears to be limited. Although sylimarin from *S. marianum* extracts alleviates Cd-induced stress by enhancing antioxidant defenses, reducing ROS accumulation and lipid peroxidation, improving photosynthesis, and restoring growth and hormonal balance in maize [109].
*Taraxacum officinale* leaves, flowers, and fruits	Chicoric acid, chlorogenic acid, luteolin, quercetin glycosides [110]	*Taraxacum officinale* extracts possess antioxidant properties [111]. *T. officinale* root extract increased shoot biomass in strawberry plants (up to ~48% in cv. ‘Albion’) and promoted root development parameters (e.g., length and architecture), indicating a biostimulant effect dependent on plant genotype [112].
*Urtica dioica* leaves	2-hydroxycinnamic acid, 3,4-dihydroxybenzaldehyde, 4-hydroxycoumarine, 4-methylumbelliferone hydrate, alpha-bisabolol, alpha-bisabolol acetate, alpha-farnesene, alpha-pinene, angelic acid, azulene, bisabolol oxide a, caffeic acid, catechol, chamazulene, ethyl protocatechuate, and p-coumaric acid [113]	*U. dioica* extracts possess antifungal properties and antioxidant properties. Foliar application of extract improved grapevine physiological performance by enhancing photosynthetic activity, increasing photosynthetic pigments, starch, and soluble sugars, and reducing lipid peroxidation, indicating mitigation of oxidative stress and overall improvement in plant productivity and yield under field conditions [114,115].
*Zingiber officinale* rhizome	Gingerols (6-shogaol, 6-gingerol, 8-gingerol, 10-gingerol), ginger phenylpropanoids [116]	Ginger extract reduced lipid peroxide levels and increased antioxidant enzyme activities in maize seedlings grown under lead, cadmium, and boron stress, indicating mitigation of metal-induced oxidative stress [117].

## Data Availability

No new data were created or analyzed in this study.

## Abbreviations

The following abbreviations are used in this manuscript:

APX	Ascorbate peroxidase
CAT	Catalase
DHAR	Dehydroascorbate reductase
GPX	Glutathione peroxidase
GR	Glutathione reductase
GSH	Glutathione
LED	Light Emitting Diode
MDA	Malondialdehyde
PAR	Photosynthetically Active Radiation
PRX	Peroxidase
PrxQ	Peroxiredoxin Q
ROS	Reactive oxygen species
SOD	Superoxide dismutase
